# Assessing the growth kinetics and stoichiometry of *Escherichia coli* at the single‐cell level

**DOI:** 10.1002/elsc.202100157

**Published:** 2022-05-06

**Authors:** Katharina Smaluch, Bastian Wollenhaupt, Heiko Steinhoff, Dietrich Kohlheyer, Alexander Grünberger, Christian Dusny

**Affiliations:** ^1^ Department of Solar Materials – Microscale Analysis and Engineering Helmholtz‐Centre for Environmental Research – UFZ Leipzig Leizpig Germany; ^2^ Microscale Bioengineering IBG‐1: Biotechnology Forschungszentrum Jülich GmbH Jülich Germany; ^3^ Multiscale Bioengineering Faculty of Technology Bielefeld University Bielefeld Germany

**Keywords:** *Escherichia coli*, growth, microfluidics, single‐cell analysis, specific growth rate, yield coefficient

## Abstract

Microfluidic cultivation and single‐cell analysis are inherent parts of modern microbial biotechnology and microbiology. However, implementing biochemical engineering principles based on the kinetics and stoichiometry of growth in microscopic spaces remained unattained. We here present a novel integrated framework that utilizes distinct microfluidic cultivation technologies and single‐cell analytics to make the fundamental math of process‐oriented biochemical engineering applicable at the single‐cell level. A combination of non‐invasive optical cell mass determination with sub‐pg sensitivity, microfluidic perfusion cultivations for establishing physiological steady‐states, and picoliter batch reactors, enabled the quantification of all physiological parameters relevant to approximate a material balance in microfluidic reaction environments. We determined state variables (biomass concentration based on single‐cell dry weight and mass density), biomass synthesis rates, and substrate affinities of cells grown in microfluidic environments. Based on this data, we mathematically derived the specific kinetics of substrate uptake and growth stoichiometry in glucose‐grown *Escherichia coli* with single‐cell resolution. This framework may initiate microscale material balancing beyond the averaged values obtained from populations as a basis for integrating heterogeneous kinetic and stoichiometric single‐cell data into generalized bioprocess models and descriptions.

**Abbreviations**
*E. coli*
: *Escherichiacoli*
GFP: Green fluorescent protein
JUNN: Jülicher U‐Net Neural Network Tool kit
OD600: Opticaldensity at 600 nm
PDMS: Polydimethylsiloxane

## 1 INTRODUCTION

In biotechnology, microbial cells can be utilized as living catalysts to perform chemical reactions and transform substrates into valuable chemical products [1]. In contrast to chemical catalysts and enzymes, a living cell's transformation performance and efficiency as a catalyst are decisively determined by its physiology and the type of connection that links the chemical reaction and cell physiology [2]. The cell feeds reactions with cofactors as energetic drivers. In fermentative bioprocesses, molecules from the central metabolic network are withdrawn as the desired chemical product [3]. For critical reactions with high enzyme turnover, the compartmentalized intracellular environment stabilizes the reaction or repairs and resynthesizes the enzymes that perform the desired reaction [4, 5]. Transport of substrate and product molecules across the cell membrane has to be facilitated by the cell [6]. All mentioned processes require energy or tap organic molecules of the cell's metabolic network [7, 8, 9].

As can be seen, knowledge of the cell's physiology is mandatory to develop efficient and competitive bioprocesses [10]. Pivotal descriptors for cell physiology in a bioprocess context are cell growth kinetics [11, 12, 13, 14], the specific rate of substrate uptake [15, 16], and the corresponding conversion stoichiometry of the energy and carbon‐containing substrate into biomass and catalytic products [17, 18]. Over decades, growth experiments with populations have been developed and performed to quantify these parameters [19, 20, 21]. Such experiments require standardized and straightforward equipment such as spectrophotometers for growth analysis and chemical assays or HPLC analytics for substrate and product quantification and are broadly available.

Based on the measured state variables and kinetics, material and energy balancing can be applied and used to identify the efficiency of the biocatalyst. These principles have become the basis of modern biotechnology and biochemical engineering sciences. However, these principles cannot be applied for single‐cell cultivation and analysis at a single‐cell scale, despite the field's significantly growing importance for biotechnological applications [22]. Driven by novel microscale cultivation approaches that grant excellent control over the extracellular environment, phenomena like gene expression, or growth heterogeneity have been broadly studied in single microbial cells [23]. Due to the analytical challenges at the single‐cell level, optical analytics based on cell‐segmentation and fluorescent markers or biosensors are still the analytical tools of choice for obtaining kinetic data of single cells – but novel approaches to access basic parameters such as the specific growth rate based on the cells dry mass increase, are still at its infancy [24, 25, 26, 27, 28, 29].

**PRACTICAL APPLICATION**

The present integrated technological and analytical framework brings the quantitative principles of biochemical engineering to single‐cell scales. Our framework combines the latest microfluidic cultivation technologies and analytics, enabling us to determine cell dry mass, specific cell density, specific growth rate, substrate affinity, and derive biomass yields on substrate from this data with single‐cell resolution. Access to such fundamental data on single‐cell physiology enables deciphering yet unknown cell–environment and cell–cell interactions in bioprocesses. Advanced single‐cell approaches deliver novel insights regarding population heterogeneity; a prerequisite of more accurate models of productive (sub) populations. Closing material balances at the microscale becomes possible now. The demonstrated framework is adaptable to other cell types relevant for bioprocesses.

The present study demonstrated a combined workflow that brings the basic concepts of quantitative physiology for the first time to the single‐cell level. At the core of this study, we established (i) quantitative phase imaging as a novel and quantitative tool that grants access to the individual microbial cells’ mass and dry matter density. (ii) The obtained data sets were then used to determine specific growth kinetics and biomass yield coefficients in novel picoliter batch reactors at defined amounts of substrate. (iii) Growth experiments under defined substrate concentrations were performed in microfluidic chemostat cultivations to characterize whole‐cell substrate affinity. Our study focused on analyzing *Escherichia coli* cells grown on glucose as sole energy and carbon substrate as a well‐studied biological system with high relevance for biotechnological applications. The employed microfluidic cultivation systems have been extensively characterized and represent state‐of‐the‐art technologies [30, 31, 32]. The systems were chosen based on their specific properties to enable the characterization of microbial physiology under the required different environmental conditions: (i) a microfluidic batch reactor to link biomass synthesis and substrate availability, (ii) a continuous microfluidic system to apply defined and steady substrate concentrations for substrate affinity, and (iii) solid growth support to mimic the early stages of shake flask cultivation.

By integrating the data of our developed concept, we approximated a material balance based on the kinetics and stoichiometry of growth in *E. coli* for the first time at the single‐cell level.

## 2 MATERIALS AND METHODS

### 2.1 Bacterial strain, growth media, and pre‐cultivation

In this study, we decided to investigate the growth kinetics and stoichiometry of the well‐known and well‐characterized *E. coli* MG‐1655. For improving visualization of the strain with fluorescence microscopy, the strain was transformed with the plasmid pVWEx1‐gfpUV to synthesize a green fluorescent protein (GFP) variant. GFP gene expression could be induced via IPTG addition. Shake flask experiments revealed that GFP gene expression did not influence the specific growth rate of the specific strain MG1655 (data not shown). Cultivations were performed according to published M9 medium recipes [33, 34, 35]. M9 medium contained (per liter of distilled water): 8.4 g Na_2_HPO_4_·2H_2_O, 3 g KH_2_PO_4_, 1 g NH_4_Cl, 0.5 g NaCl, 15.0 mg CaCl_2_·2H_2_O, 0.50 g MgSO_4_·7H_2_O, supplemented with trace elements 100 mg FeSO_4_·7H_2_O, 100 mg MnSO_4_·H_2_O, 10 mg ZnSO_4_·7H_2_O, 3.71 mg (NH_4_)_6_Mo_7_O_24_, 3.13 mg CuSO_4_·5H_2_O, 2.47 mg H_3_BO_3_, 0.2 µg NiCl_2_·6H_2_O, 50 µg Kanamycin, and 1% w/v d‐glucose. Overnight pre‐cultures of *E. coli* MG1655 pVWEx1gfp‐UV were inoculated from glycerol stock in 10 mL M9 medium in 100 mL flasks on a rotary shaker at 200 rpm. Cells from the overnight culture were transferred to inoculate the second culture with a starting OD_600_ of 0.1. The cells were prepared for follow‐up cultivation at OD_600_ of around 1.

### 2.2 Population cultivation setups

The present study employed the following three microscale cultivation systems: (i) agarose pad, (ii) microfluidic Mother Machine cultivation channel, and (iii) microfluidic picoliter batch reactor.

#### 2.2.1 Agarose pad

For the estimation of single‐cell dry matter density of *E. coli*, agarose pads were prepared similar to the published protocol [36]. In more detail, 20 mL of freshly prepared M9 medium as described in Subsection 2.1 was supplemented with low‐melt agarose (1.5% w/v) and gently warmed until the agarose was fully dissolved. After a short cool down, between 10^−6^% w/v and 1% w/v d‐glucose was added to the M9 agarose melt. Then, 300 µL of M9 agarose melt was transferred onto a sterile glass coverslip (Ø 18 mm) and immediately covered with a second sterile coverslip forming a glass‐agarose‐glass sandwich. As soon as the agarose layer fully solidified, the top coverslip was carefully removed. Finally, the agarose surface was irrigated with five droplets á 0.5 µL cell suspension at OD_600_ = 0.08 and sufficient distance to each other to prevent a merging of the droplets. Afterwards, the liquid was allowed to spread and dry for 15 min. The agarose pad was then placed upside‐down in a µ‐dish (35 mm µ‐dish glass bottom, Ibidi, Germany) which was filled with respective M9 medium preventing evaporation. Bacterial growth analysis by time‐lapse microscopy and cell dry mass monitoring by quantitative phase imaging were performed on identical samples. Therefore, the µ‐dish was mounted onto the microscope stage and cultivations were performed at 37°C for 24 h.

#### 2.2.2 Mother Machine

Polydimethylsiloxane (PDMS) based microfluidics required special silicon/SU8 mold wafers, which were fabricated by SU8 photolithography and subsequent PDMS molding (Supplemental Information [SI]).

For substrate affinity estimation, Mother Machine growth channels were adapted from Lindemann et al. using a channel width of 1.5 µm and height of 0.85 µm [37].

The geometry provides a quasi‐one‐dimensional growth channel to restrict cell proliferation along one axis. The Mother Machine growth channels are arranged between two parallel supply channels. Supply channel was perfused with fresh medium continuously ensuring a constant cultivation condition and washing out of metabolic by‐products simultaneously.

#### 2.2.3 Picoliter batch reactor

Microfluidic batch cultivations for growth and biomass yield analysis were performed according to Kaganovitch et al. [32]. For statistically valid mass balancing, however, each growth chamber is additionally connected to a medium reservoir resulting in a total cultivation volume of 606 pL. Furthermore, only a single inlet was implemented for each cultivation site, allowing a more efficient isolation (batch mode). The device features six cultivation rows, each consisting of an array of 33 individual cultivation chambers, each 60 µm × 100 µm × 1 µm (width, length, and height), enabling monolayer cell growth. The medium reservoir and the cultivation chamber are separated by a thin double grid PDMS structure, allowing medium exchange between the shallow growth region and the deeper reservoir, but preventing cells from entering the medium reservoir. A particular concern in picoliter batch cultivation for single‐cell analysis is the permeability of PDMS to gases and vapors [38], which leads to evaporation of the cultivation medium. Therefore, each cultivation channel is accompanied by two parallel side channels continuously flushed with water. Furthermore, the whole chip is covered with a water layer, which comprises an additional measure to prevent evaporation of the medium.

### 2.3 Microfluidic cultivation experiments

#### 2.3.1 Agarose pad

Single‐cell cell agarose pad cultivations were performed according to the protocol of Dusny et al. [36]. Cultures were grown with extracellular glucose concentrations in the range of 10^−6^% w/v–10% w/v d‐glucose until exponential phase with a starting OD_600_ of 0.1 was washed once and diluted to a cell density of OD_600_ = 0.08 and applied to an agarose pad. To prevent thermal drifts and ensure cell adaptation, the agarose pad was placed in the prewarmed 37°C in‐house fabricated dish holder inside the automated microscope incubator for 1 h before following time‐lapse imaging with ZEN blue (v 3.3, Carl Zeiss Microscopy GmbH, Germany) and PHAST (v 2.4.1, Phaesics, France) in parallel.

#### 2.3.2 Mother Machine

For substrate affinity estimation, cells from the overnight culture were transferred to inoculate a second culture with a starting OD_600_ of 0.1. The Mother Machine chips were manually inoculated with a cell suspension of an OD_600_ of 1, using a 1 mL disposable syringe. The seeding of cells into the growth channels was a random process, achieved by manually applying an alternating pressure to the syringe. The induced pulsating flow into the microfluidic chip pushes the cells into the channels. After seeding, the microfluidic chip is connected to computer‐controlled pressure fluid pumps (Flow EZ Pressure Controller, Fluigent, France). Inlet pressure of 150 mbar is applied for constant medium supply. The cells were equilibrated with M9 medium containing 1% w/v d‐glucose for 2 h [39]. After equilibration, growth studies were carried out in technical and biological replicates, containing 0% w/v–5% w/v d‐glucose at 37°C. Here, every medium was additionally sterile filtered to prevent channel clogging during microfluidic experiments.

#### 2.3.3 Picoliter batch reactor

For estimation of growth and biomass yield in picoliter batch experiments, culture preparation was performed as described before (Mother Machine). At OD_600_ = 1.1 mL of the cell suspension was washed twice (5000 g, 10 min) with M9 media containing the same glucose concentration as the following growth experiment, ranging between 10^−4^% w/v and 0.05% w/v glucose at 37°C. First, microfluidic side channels and water reservoir were perfused with sterile deionized water between 40 and 90 mbar. Before cell inoculation, the medium reservoirs were filled with M9 medium ensuring well‐defined starting conditions. Afterward, the cell suspension was diluted to an OD_600_ of 0.1 and flushed into the microfluidic chip by manually applying pressure to a 1 mL syringe. This flow causes single cells to be randomly flushed into the picoliter batch chambers. After inoculation, the medium channels were flushed with fresh M9 medium for 15 min at constant flow of 200 nL min^−1^ (nemeSYS syringe pumps, Cetoni GmbH, Germany). Before starting the experiment, the medium channels were emptied by applying 450–600 mbar of compressed air (Flow EZ Push‐Pull, Fluigent, Germany) to isolate the individual cultivation chambers from each other.

### 2.4 Time‐lapse imaging

#### 2.4.1 Agarose pad

For the determination of single‐cell dry mass, cell length, and width, time‐lapse microscopy was performed using an inverted live‐cell microscope from Zeiss (Axiocam 503, Carl Zeiss Microscopy GmbH, Germany), including a temperature incubator (incubator, XLmulti S2 Dark, Pecon., Germany) mounted on the microscope stage. The system was equipped with the camera wavefront sensor c‐mounted (SID‐4‐sC8 sCMOS, Phaesics, France). The microscope set up included a 100× oil immersion objective, 60× optovar (magnification 160×, 0.045 µm px^−1^), and an automated defined focus system (Definite Focus 3, Carl Zeiss Microscopy GmbH, Germany) to compensate thermal drifts during long term imaging time‐lapse microscopy. Time‐lapse images were recorded in parallel with ZEN blue (v 3.3, Carl Zeiss Microscopy GmbH, Germany) and PHAST (v 2.4.1, Phaesics, France) every 5 min for 24 h. Before acquisition, a modified Köhler illumination was conducted as described in SID4BIO user's manual for quantitative phase imaging. The image acquisition process was performed as described before [40].

#### 2.4.2 Mother Machine

For time‐lapse imaging, an inverted automated microscope from Nikon (Nikon Eclipse Ti2, Nikon, GmbH, Germany) equipped with temperature incubator (Cage incubator, OKO Touch, Okolab S.R.L., Italy) was used. The microfluidic device was mounted in an in‐house fabricated chip holder and placed inside the incubator. Additionally, the setup was equipped with a 100× oil objective (CFI P‐Apo DM Lambda 100× Oil, Nikon GmbH, Germany), DS‐Qi2 camera (Nikon camera DS‐Qi2, Nikon GmbH, Germany), and an automated focus system (Nikon PFS, Nikon GmbH, Germany) to compensate thermal drift during long‐term microscopy. Eighty cultivation chambers were selected manually for each experiment and were monitored using the NIS‐Elements Imaging Software (Nikon NIS Elements AR software package, Nikon GmbH, Germany). Time‐lapse images were recorded every 5 min.

#### 2.4.3 Picoliter batch reactor

The picoliter batch device was placed on a time‐lapse microscope setup in an incubation chamber (Temp Controller 2000‐2, PeCon, Germany). Phase contrast images were acquired every 20 min. The basic microscope setup was the same as used for the Mother Machine experiments.

### 2.5 Data analysis

#### 2.5.1 Agarose pad for cell dry mass and density determination

Cell dry mass analysis was carried out using SID4BIO‐1031 (v 2.4.4., Phaesics, France), including background subtraction according to Aknoun et al. [40]. An image stack for all time points of interest of a selected glucose concentration was created and used for cell segmentation. Single‐cell segmentation was carried out in manual mode for at least 50 single cells over a cultivation time of 6 h to obtain the optical thickness of each cell. Lengths and widths of every cell were measured individually for subsequent cell volume calculations.

#### 2.5.2 Mother Machine cultivation for substrate affinity determination

For the determination of cell growth kinetics and substrate affinities under the given substrate conditions, live‐cell image sequences were analyzed using the open‐source software Fiji 1.52 [41]. For determining single‐cell division events, one mother cell was selected whose offspring was present until the end of the measurement. Based on the growth of this cell line, it can usually be guaranteed that at least 50 single‐cell division events are present. If a selected glucose concentration resulted in minimal growth, we would differentiate between two events: (i) Multiple cell lines are summed and quantified if the number of generations is at least 4 but has less than 50 division events, or (ii) the total number of generations is less than 4, then it is defined as no growth.

Doubling times of each offspring of the selected cell line will be determined after the first division event through manually marking by using the integrated multi‐point function of Fiji to ensure a frame‐independent tracking of temporally asymmetric divisions depending on the generation time. The respective mean values for doubling times and growth were determined using the geometric mean [42], which is less susceptible to the influence of outliers. Furthermore, the mathematical standard deviation, defined here as growth heterogeneity, was determined by weighted standard deviation. Here, extreme outliers like two consecutive division events were weighted with 0 because this event reveals regressive problems [43]. The substrate affinity *K*_S_ was estimated graphically while assuming a normal distributed database by determining the slope of the sigmoidal regression from the resulting logarithmical plot using OriginPro (OriginPro 2020 9.7.0.188; OriginLab Corporation).

#### 2.5.3 Picoliter batch reactor cultivatons for the determination of specific growth rates and biomass on substrate yield coefficients

The live‐cell image sequences were analyzed using the open‐source software Fiji 1.52 [41]. Images had to be pre‐processed manually, including brightness correction, image alignment, and definition of the region of interest. Semi‐automatic cell segmentation on image data stacks was achieved using in‐house developed deep learning cell segmentation tool “Jülicher U‐Net Neural Network Tool kit” (JUNN). JUNN segments cells in each image to derive the cell width, length, and projected cell area. The biomass yield coefficient was then calculated for each chamber individually. For this purpose, the final total cell volume was first calculated using the projected total cell area and the known chamber height. The final biomass was then calculated using the determined total cell volume and specific cell density provided through quantitative phase imaging. The ratio between the biomass formed and the initial amount of glucose in the cultivation vessel was then calculated for the substrate‐to‐biomass yield coefficient.

### 2.6 Dynamic modeling

The dynamic modeling was based on the obtained single‐cell data and performed with Berkeley Madonna (V 8.3.18, Berkeley Madonna). All statistical tests were performed with SPSS (IBM Corp. Released 2020. IBM SPSS Statistics for Windows, Version 27.0. Armonk, NY: IBM Corp).

The model was based on a batch reactor material balance and the respective set of equations for describing the kinetics of the system. A Monod‐based model was used to describe the relationship between extracellular substrate concentration and the specific growth rate of the cells. Specific substrate uptake and by‐product synthesis rates were calculated based on the experimentally determined values. Physiological heterogeneities were implemented into the model as the observed distribution functions of the substrate affinity *K*_S_.

## 3 RESULTS AND DISCUSSION

**FIGURE 1:**
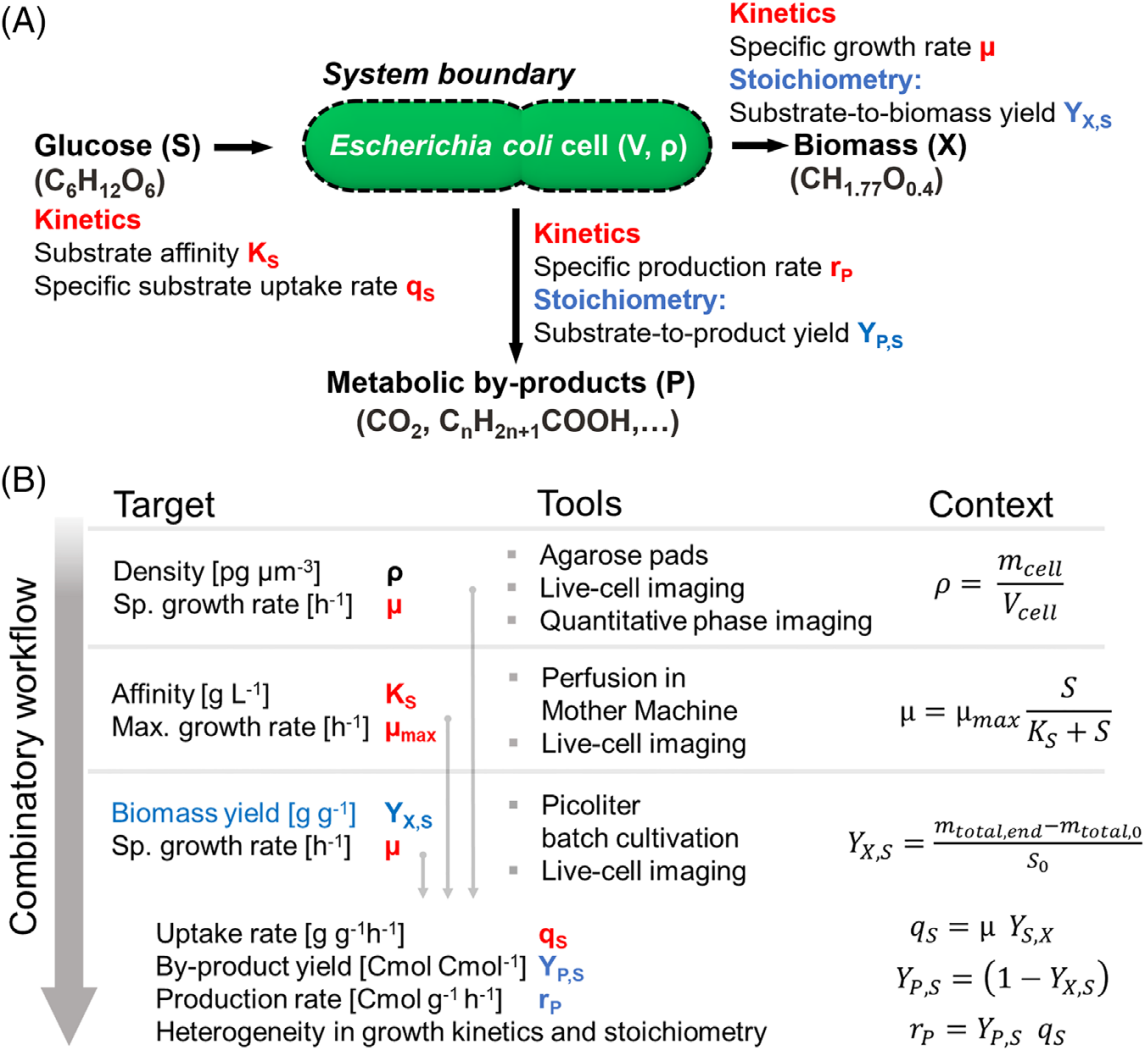
(A) Kinetics (red) and stoichiometric coefficients (blue) that describe the growth of *E. coli* on glucose. (B) Combined workflow to acquire the required parameters at the microscale. The left column gives the target parameters, while the middle column describes the microfluidic cultivation methods and the corresponding analytics. The workflow is built on and connected by the corresponding mathematical expressions given in the right column

Our study's experimental design and workflow development were dictated by the mathematical description of growth kinetics and stoichiometry in glucose‐grown *E. coli* cells. The parameters to be acquired are summarized in Figure 1A. The combinatory workflow is visualized in Figure 1B, denoting the physiological target parameters, the respective tools for their acquisition, and their mathematical links. The elemental composition of biomass in *E. coli* MG1655 was based on data from our own laboratories and previously published literature data [18].

For single‐cell level material balancing, the cell dry weight and specific dry matter densities constitute fundamental parameters, as the specific substrate uptake rate *q*_S_ [g g^−1^ h^−1^] and specific product formation rate *r*_P_ [g g^−1^ h^−1^] can be determined by correlating biomass yield *Y*_X,S_ [g g^−1^] or product yield *Y*_P,X_ [g g^−1^] with the specific growth rate μ [h^−1^] (see Equations 1 and 2):

(1) $$q_{s}=\;-\;\frac{dS}{dt}=\;-\frac{\mu }{Y_{X,S}}\;$$

(2) $$r_{P}=\frac{dP}{dt}\;=\;\mu \;Y_{P,X}$$

As the specific growth rate, substrate‐to‐biomass yields, and product‐per‐biomass yields are specific to the cell concentration expressed in dry matter per volume, the cell dry mass must be accessible in microfluidic cultivations to perform adequate quantifications of cellular physiology. Based on the knowledge of dry weight, projected cell area, and volume, the data acquired by live‐cell imaging could be transformed into dry mass densities. Future studies will enable to determine substrate and product formation rates in microcultivations, relying on accurate dry mass measurements of cells to enable kinetic comparisons of populations and single cells [44].

### 3.1 Optical dry mass and dry mass density determination of single *E. coli* cells

Non‐optical approaches exist to determine the dry mass of individual cells, but most approaches are not practical to implement into microfluidic cultivations concepts that allow the cultivation of cells at defined environmental conditions simultaneously [45, 46, 47]. Therefore, we established the principle of optical mass imaging for dry weight determination of single *E. coli* cells in our study.

Non‐invasive optical profiling of single‐cell dry masses was performed via quantitative phase imaging. The measurement principle of quantitative phase imaging relies on quantifying phase differences resulting from light passing through a biological object, which directly correlates to the dry mass of the biological object. For a comprehensive overview of the optical dry mass measurements, we refer to the excellent review of Zangle and Teitell [48]. The sensitive and straightforward method of quantitative phase imaging was applied to map the conditions‐dependent absolute masses of single *E. coli* cells in microfluidic cultivations with sub‐pg resolution. In control experiments, we optically weighed standardized latex particles of known dry mass density with dimensions similar to the dimensions of *E. coli* cells via quantitative phase imaging before the actual biological experiments were carried out to validate the method for dry mass determination of cells (see SI). The uncertainty for latex particle mass density determination was determined to be 1.05 ± 0.04 pg µm^−3^, which is excellent compared to population‐based methods for cell dry matter determination based on spectrophotometry combined with weighing of dried biomass samples.

**FIGURE 2:**
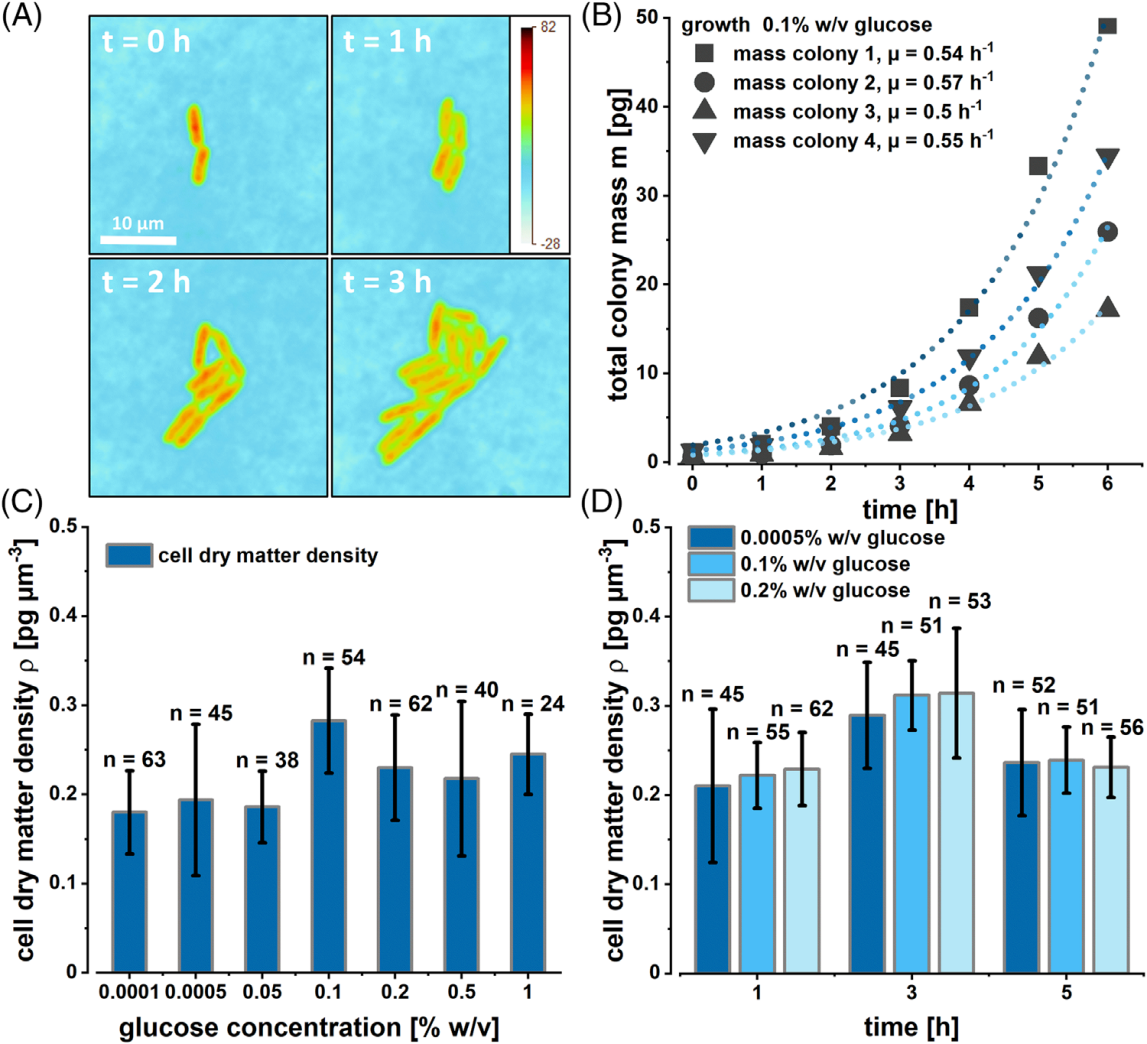
(A) Time‐resolved mass imaging of a single *E. coli* colony based on quantitative phase imaging. The color bar in the upper right corner denotes the optical thickness. (B) Growth of four individual *E. coli* colonies at a glucose concentration of 0.1% w/v followed with quantitative phase imaging. (C) Determined cell dry matter densities ρ of individual *E. coli* cells grown at different extracellular glucose concentrations after incubation on agarose pads. (D) Dynamics of single‐cell dry matter densities tracked over a total cultivation time of 5 h. *n* refers to the number of individual cells that have been analyzed per glucose concentration or time point, respectively

As the microscale cultivation method of choice, we used agarose pads since the very high medium to biomass ratio allows monitoring single‐cell growth under steady‐state extracellular substrate conditions. Agarose pads enabled monolayer cell growth, which was necessary for the optical analysis of single *E. coli* cells via quantitative phase imaging. The dynamics and heterogeneity of individual *E. coli* cells in response to extracellular glucose concentrations are still largely unexplored, and our initial analysis focused on quantifying cell masses during exponential growth at different extracellular glucose concentrations. *E. coli* cells in the early exponential growth phase were taken from low‐density shake flask cultivations at different glucose concentrations. This inoculum was spread on the agarose pads with the same respective glucose concentration as the shake flask cultures. Cells and the resulting microcolonies grew exponentially at all applied glucose concentrations, and cell dry masses could be measured over several hours until confluence of the respective microcolonies was reached. As can be seen from the phase images, the mass resolution of quantitative phase imaging is sufficient to resolve small mass heterogeneities within single cells (see Figure 2A). The lighter cell envelope could be clearly distinguished from the dense cytosol of the cells. The obtained glucose‐dependent specific growth rates of individual microcolonies based on quantitative phase imaging measurements ranged from µ = 0.19 h^−1^ to µ = 0.61 h^−1^ (see Figure 2B). They were similar to the specific growth rates of populations grown in shake flasks and the other applied microfluidic cultivation approaches at the respective extracellular glucose concentrations. For all further microscale cultivation experiments of the developed combined workflow, the specific growth rate of the cells at given glucose concentrations was aligned to ensure comparative physiology of the cells in all applied microscale cultivations. By simultaneously using quantitative phase imaging measurements and morphometric measurements of cell dimensions via brightfield imaging, dry mass *m*_cell_ [pg] and the volume *V*_cell_ [µm^3^] could be correlated to obtain the dry mass density *ρ* [pg µm^−3^] of individual cells (see Equation 3). Please see the SI file for a detailed description of the single‐cell volume calculations.

(3) $$\rho \;=\frac{m_{\mathrm{cell}}}{V_{\mathrm{cell}}}\;$$

Averaged cell dry mass densities $\bar{\rho }$ ranged from 0.13 to 0.28 pg µm^−3^ and displayed a pronounced heterogeneity (see Figure 2C, D). However, dry mass densities of the individual cells remained virtually constant during exponential growth for extended cultivation periods of up to 5 h (see Figure 2D). This finding implies that cellular control mechanisms result in an individualized dry mass density homeostasis in *E. coli* cells [49, 50]. The averaged dry mass densities slightly increased with along extracellular glucose concentrations (confirmed by statistical *t*‐test with *p* = 0.042 and hence *p* < 0.05). This observation implies further studies to reveal a possible correlation between dry mass density, extracellular glucose‐concentration, and growth. The obtained data was sufficient for the present study to follow the developed workflow toward microscale material balancing.

### 3.2 Analysis of whole‐cell kinetics of individual *E. coli* cells in physiological steady‐state at constant substrate conditions

The rate of growth of a cell is governed by the affinity of the substrate‐transporting enzyme system toward the substrate itself. The Monod equation mathematically describes the correlation between the specific growth rate and the specific substrate uptake rate (see Equation 4). The main kinetic parameters in this equation are the half‐velocity (Monod) constant *K*_S_ as a measure for the affinity of the cells toward the substrate, as well as the maximal achievable specific growth rate μ_max_ under non‐substrate limiting conditions

(4) $$\mu =\mu_{\max}\frac{S}{K_{S}+S}$$

**FIGURE 3:**
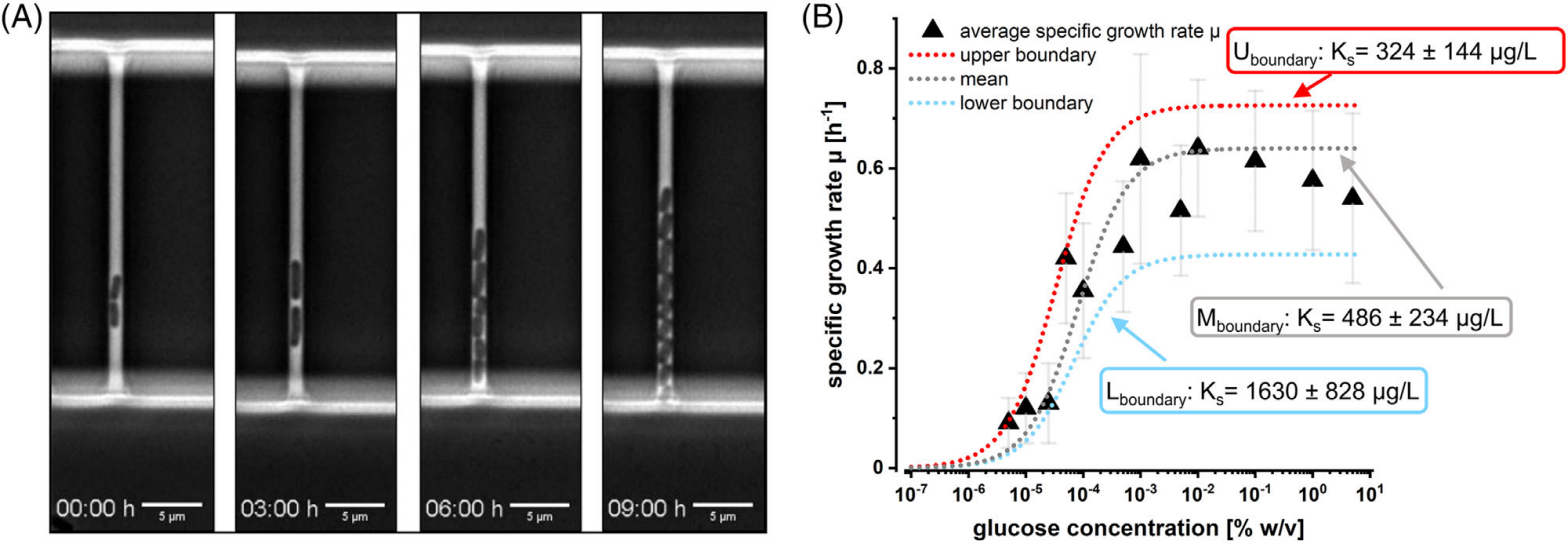
(A) Time‐lapse images of *E. coli* cells grown under constant glucose concentration in the Mother Machine. (B) Glucose‐dependent maximum specific growth rates of individual *E. coli* cells grown in microfluidic perfusion cultivations (Mother Machine). The black line denotes the fit of the Monod equation to the dataset, while the red and blue lines give the upper and lower boundaries of the mathematical fit, respectively

It was, therefore, necessary to determine μ_max_ and *K*_S_ of the target *E. coli* in microscale cultivations. For lab‐scale experiments, continuous cultivations in substrate‐limited chemostats are the measure of choice to quantify these parameters [51]. We employed microfluidic continuous perfusion bioreactors for our study based on the Mother Machine cultivation principle (see Figure 3A). This cultivation principle enabled controllable and steady glucose conditions for linking the measured single cell‐specific growth rates with the applied glucose concentration. The specific growth rates μ of the individual *E. coli* cells were determined by cell segmentation on image‐data stacks derived from automated time‐lapse microscopy. The cells were grown at steady glucose concentrations ranging from 0% w/v to 10% w/v, yielding a Monod‐type response of the specific growth rates of individual cells, with an observed maximal specific growth rate μ_max_ = 0.66 h^−1^ (see Figure 3B). A mathematical fit of the obtained data yielded an average *K*_S_ value of 486 ± 234 µg L^−1^ for our particular *E. coli* strain grown on glucose, with an upper boundary *K*_S_ = 324 ± 144 µg L^−1^ and a lower boundary of *K*_S_ = 1630 ± 828 µg L^−1^. The large standard deviations arise from a pronounced heteroegenity in division timing of the cells. However, small deviations in the detection precision of the division also resulted in high deviations in the determined growth rate. This aspect was compensated by the sheer number of individual cells that have been tracked and counted. The determined averaged Monod constant for glucose‐grown *E. coli* cells is well comparable to literature data, which for example, state a population *K*_S_ value of 366 ± 36 µg L^−1^ [51]. However, while this result was expected and further validated the applicability of the developed microscale cultivation concept, the obtained data deliver first insights into potential heterogeneities in the specific glucose uptake capacity of individual *E. coli* cells. As the magnitude of heterogeneity, even under such constant growth conditions, is comparable to the determined heterogeneity of single‐cell dry mass (∼±40%), one might speculate that the cells’ glucose uptake and processing capacity are linked to their catalytically active dry mass. Although the observed relationship between the dry mass of single cells and their catalytic activity was not the target of this study, it will be analyzed by combining microfluidic perfusion cultivations with quantitative phase imaging in the future. Statistical tests of the applied sigmoidal mathematical fits consistently resulted in an *R* ^2^ > 0.95 for mean values, as well as the upper and lower boundaries.

### 3.3 Determination of substrate‐to‐biomass yield coefficients in picoliter batch reactors

After determining the kinetics of growth and substrate uptake in *E. coli*, the stoichiometry of growth was focused on closing the microscale material balance. As a practical implementation of substrate‐to‐biomass yield measurements, the absolute dry mass of synthesized biomass (*m*_total,end_ − *m*_total,0_) at a known available amount of added glucose *S*_0_ within the picoliter batch system can be used to calculate *Y*_X,S_ (see Equation 5).

(5) $$Y_{X,S}=\frac{m_{\mathrm{total},\;\text{end}}-m_{\mathrm{total},0}}{S_{0}}\;$$

**FIGURE 4:**
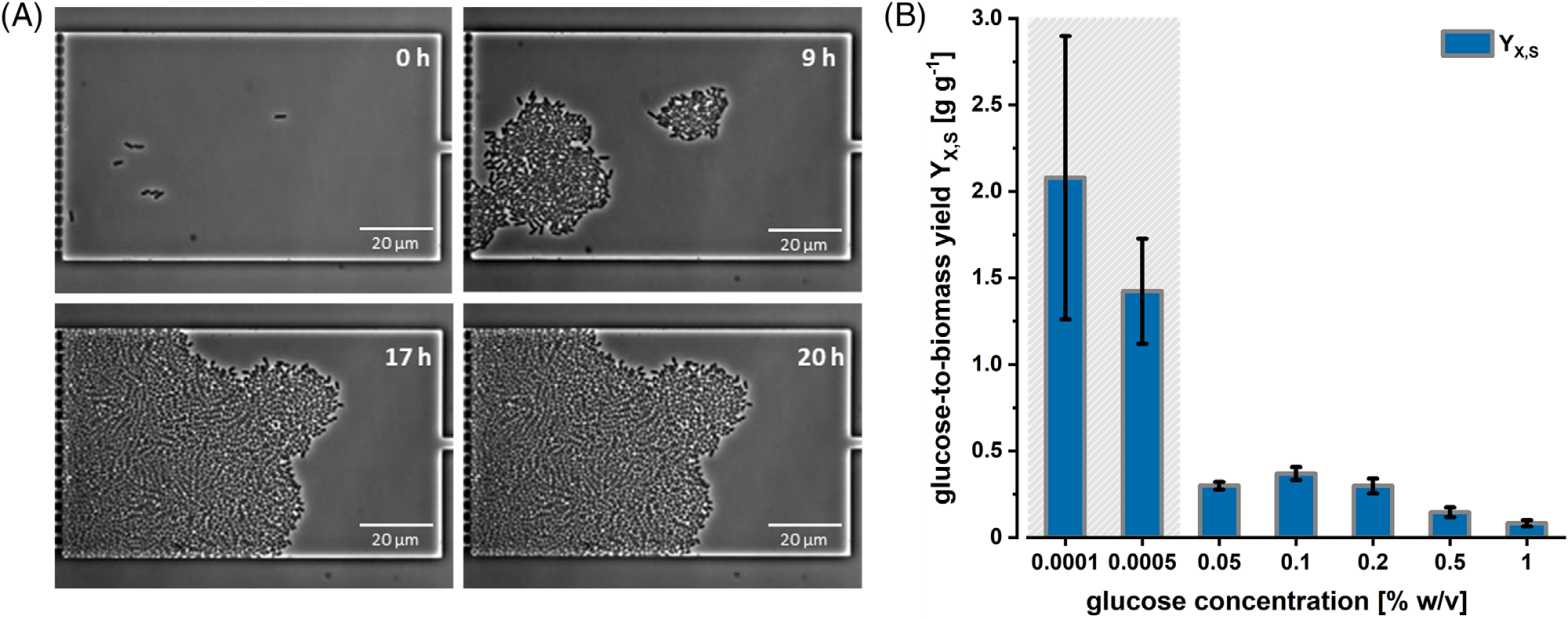
(A) Exemplary *E. coli* colony grown in a picoliter batch reactor monitored by time‐lapse imaging. (B) Substrate‐to‐biomass yields coefficients *Y*_X,S_ determined at different glucose concentrations

To measure the glucose‐to‐biomass yield in *E. coli*, the novel concept of the picoliter batch reactor was employed [32]. The batch reactor is based on the previously developed principle of parallelized picoliter growth chambers, but with sealable reactor chambers and a substrate reservoir attached to each chamber [36, 52, 53]. As the volumes of the reaction chambers and substrate reservoirs were known, the total amount of available substrate could be accurately adjusted. Cells from exponentially growing pre‐cultures were seeded into the respective growth chambers with low starting biomass (few cells) at different initial glucose amounts *S*_0_. Cell growth was followed by analyzing time‐lapse image data determining the specific growth rates of the microcolonies inside the picoliter batch reactor chambers (see Figure 4A). Upon reaching the stationary growth phase, the total cell volume was calculated from the cell number and the individual cell dimensions. The total biomass in the chambers was then calculated based on the dry mass densities determined by quantitative phase imaging at the respective glucose concentrations. Substrate‐to‐biomass yields were determined at glucose concentrations ranging from 10^−5^% w/v to 1% w/v with the corresponding standard deviations calculated based on the error propagation from the cell dry mass density calculations. The results are displayed in Figure 4B.

The determined yield coefficients *Y*_X,S_ were in the reported range of 0.35–0.5 g g^−1^ for *E. coli* at glucose concentrations of 0.05% w/v, 0.1% w/v, and 0.2% w/v comparable yields were reported for conventional laboratory‐scale cultivations [54]. Interestingly, at glucose concentrations below this range, the yields coefficients were above 1, while at concentrations above 0.2% w/v, yield coefficients below 0.25 g g^−1^.

The picoliter batch cultivations enabled us for the first time to estimate glucose‐to‐biomass yields at extremely low glucose concentrations, far below what has been accessible by lab‐scale approaches so far. At such very low cell density, the typical optical density read‐out is not sensitive enough to resolve cellular growth below 5 × 10^−5^% w/v of initial glucose. In contrast, time‐lapse microscopy enables determining cell growth of individual cells, as well as occurring growth heterogeneity.

At extremely low substrate concentrations (*S*_0_ = 10^−5^% w/v and *S*_0_ = 5 × 10^−5^% w/v), slow growth and relatively low cell numbers resulted in disperse cell monolayers. This resulted in high cultivation volume to cell number ratios and meaninglessly high glucose‐to‐biomass yields (see Figure 4B, gray area). Potential reasons for these observations might be found in glucose adsorption or glucose accumulation effects, as minor changes in glucose concentration availability result in high deviations in the determined glucose‐to‐biomass yield. The same holds for deviations in chamber height, which would also change the amounts of total available glucose inside the picoliter batch reactors. However, as such low substrate concentrations are of no practical relevance for bioprocesses, and the error caused by these effects at glucose concentrations above 0.05% w/v was estimated to be less than 1%, we did not further include the glucose‐to‐biomass yields at *S*_0_ = 10^−5^% w/v and *S*_0_ = 5 × 10^−5^% w/v in our material balance approximation.

Nevertheless, exciting effects could also be observed at high glucose concentrations (*S*_0_ = 0.5% w/v and *S*_0_ = 1% w/v). *E. coli* grew exponentially inside the picoliter batch reactors, thereby forming large and densely packed cell monolayers. Depending on the colony size and level of packing, cells along the perimeter of the colony were sufficiently supplied with substrate, whereas central cells of the colony might have become limited [55, 56]. Furthermore, growth‐induced pressure gradients caused colony compression resulting in inaccurate cell segmentation and the underestimation of the overall biomass. In the present calculations, homogenous individual cell density was assumed across the entire colony, which could be resolved by hyphenating picoliter batch cultivations with quantitative phase imaging, planned in further experiments. Increased demand by cell maintenance due to mechanical stress on the cells might have further decreased to achievable substrate‐to‐biomass yields.

However, not only technical reasons have to be taken under consideration. Another explanation for our observations might be related to the fermentation abilities of *E. coli*. Overflow metabolism in *E. coli* occurs at glucose concentrations above ∼30 mg L^−1^ [57]. At higher glucose excess, by‐product formation cannot be avoided. Andersson et al. reported for aerobic growth of *E. coli* under glucose excess an accumulation of acetic acids in concentrations which have an inhibitory effect on growth due to the surplus in energy demand imprinted by molecular measures to counteract acetate toxicity [58]. As the energy yield per molecule of metabolized glucose in fermentative pathways is much less efficient, overflow metabolism might be a possible explanation for the observed low biomass yield [59, 60]. For substrate limiting conditions, overflow metabolite production decreases, but the higher share of cell maintenance on the taken‐up glucose decreases the specific growth rate and glucose‐to‐biomass yields [61].

### 3.4 Approximating the microscale material balance and integration of single‐cell data in a dynamic culture model

**FIGURE 5:**
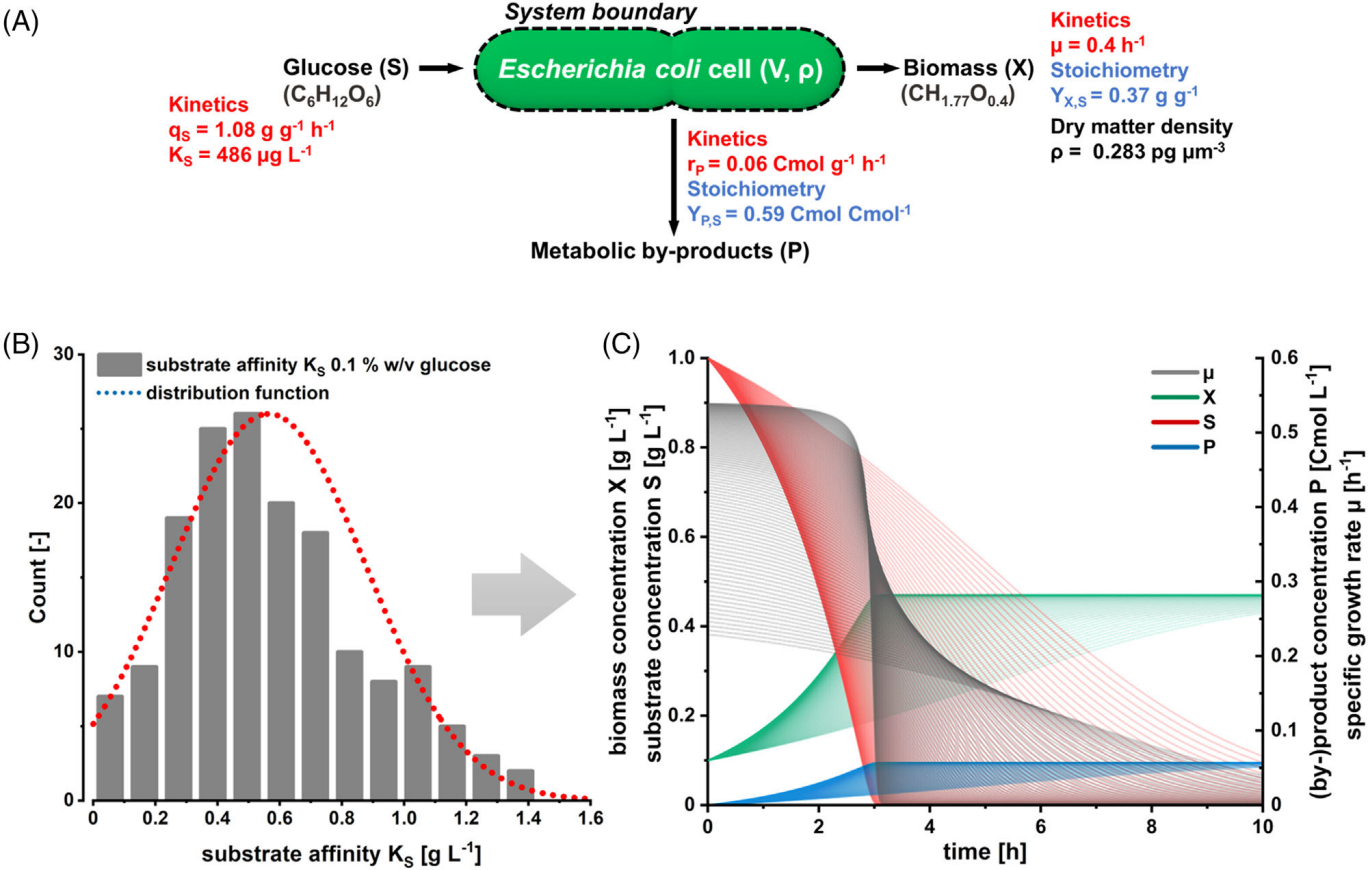
(A) Kinetic parameters and growth stoichiometry of *E. coli* grown at 0.1% w/v glucose. (B) The frequency distribution of *K*_S_ values obtained from single *E. coli* in Mother Machine cultivations. (C) The physiological space of *E. coli* growth phenotypes was obtained by including the *K*_S_ normal frequency distribution at 0.1% w/v glucose concentration into a dynamic process model

We here demonstrate a combined workflow that can be used to determine the kinetics and stoichiometry of growth for glucose‐grown *E. coli* cells. The obtained kinetic and stoichiometric parameter set, including cell dry matter concentrations, specific growth rates, the whole‐cell affinity toward glucose, as well as the glucose‐to‐biomass yield, was used to close the material balance and deduce formation kinetics and stoichiometry of carbon‐based metabolic (by‐)products such as CO_2_ or organic acids. The specific product formation rate *r*_P_ [Cmol g^−1^ h^−1^] and the glucose‐to‐product yield coefficient *Y*_P,S_ [Cmol Cmol^−1^] were determined by a carbon material balance around the cell and expressed on a Cmol‐basis as identity and stoichiometry of the by‐products could not be measured. The obtained results for cells grown at a glucose concentration of 0.1% w/v are given in Figure 5A.

As the data obtained in this study contained averaged physiological values and cell‐to‐cell heterogeneities in terms of growth kinetics and their respective frequency distributions, population behavior and the effects of cellular heterogeneity on process performance can now be linked. In a first attempt, we included data on kinetic variations of the substrate affinity *K*_S_ and the likelihood of its occurrence into a simple dynamic process model (see Figure 5B). By iterating the dynamic batch process model that was based on the measured single‐cell data, the kinetic and stoichiometric space of individual cells of can be represented in silico, demonstrating how the heterogeneous properties of single cells contribute to the cumulative overall output of a the culture (see Figure 5C).

## 4 CONCLUDING REMARKS

Formally seen, this study represents the blueprint for future biochemical engineering approaches at the microscale. For decades, microfluidic single‐cell studies had to rely on relative cell parameters, lacking numbers for kinetic and stoichiometric profiles of a single cell. We demonstrate how kinetic and stoichiometric parameters can be accessed by utilizing the individual strengths of microfluidic cultivation and analysis technologies. The access to such data also paves the way to next‐generation computational approaches for simulating population behavior, for example, individual‐based modeling. Particular emphasis is here paid to quantitative phase imaging, constituting a novel but universally usable tool to access cell mass non‐invasively with high accuracy. Although this study is demonstrated for *E. coli* only, the concept should be adaptable to any other microbial cell type that is culturable within microfluidics.

There is undoubtedly the need to intensify endeavors in quantitative single‐cell physiology. However, we are confident that the present study will bring single‐cell studies to the next level and contribute toward closing the gap between physiological studies at the single‐cell and typical shake flask and bioreactor experiments.

## NOMENCLATURE

**Table:** 

µ[h^−1^]	[h^−1^]Specific growth rate
μ_max_[h^−1^]	[h^−1^]Maximum specific growth rate
*q*_S_[g g^−1^ h^−1^]	[g g^−1^ h^−1^]Specific substrate/glucose uptake rate
*r*_p_[Cmol g^−1^ h^−1^]	[Cmol g^−1^ h^−1^]Specific production rate
*Y*_X,S_[g g^−1^]	[g g^−1^]Substrate‐to‐biomass yield coefficient
*Y*_P,S_[Cmol Cmol^−1^]	[Cmol Cmol^−1^]Substrate‐to‐product yield coefficient
*K*_S_[g L^−1^]	[g L^−1^]Apparent whole‐cell substrate affinity
*ρ*[pg µm^−3^]	[pg µm^−3^]Dry mass density
$\bar{\rho }$[pg µm^−3^]	[pg µm^−3^]Average dry mass density
*m*_cell_[pg]	[pg]Cell dry mass
*V*_cell_[µm^3^]	[µm^3^]Cell volume
*S*[g L^−1^]	[g L^−1^]Substrate/glucose concentration
*P*[g L^−1^]	[g L^−1^](By‐)product concentration

## INDICES

**Table:** 

0	value at the beginning of the cultivation
_S_	Related to substrate
_P_	Related to product
X	related to biomass
_total_	Sum of the respective value

## CONFLICT OF INTEREST

The authors have declared no conflicts of interest.

## Supporting information

Supporting Information
