# Supplementary material for: Assessing the growth kinetics and stoichiometry of Escherichia coli at the single‐cell level
Source: Eng Life Sci. 2022 May 6;23(1):e2100157. doi: 10.1002/elsc.202100157 (PMC9815083; doi:10.1002/elsc.202100157)
Supplement: Supplementary file 1 — Supporting Information [file ELSC-23-e2100157-s001.docx]

**Supplementary Information**

**Assessing the growth kinetics and stoichiometry in *Escherichia coli* at a single-cell level**

Katharina Smaluch^1,*^, Bastian Wollenhaupt^2,*^, Heiko Steinhoff^3,*^, Dietrich Kolhheyer^2^, Alexander Grünberger^3^, Christian Dusny^1,§^

**Contents**

[Chip design and wafer preparation 2](#_Toc89196434)

[Cell volume calculation 3](#_Toc89196435)

[Transformation of *E. coli* EcLYS1 (pVWEx1-gfpUV) 4](#_Toc89196436)

# **Chip design and wafer preparation**

In the following, the primary chip-processing steps and parameters are described.

Photolithography was performed under cleanroom conditions. A 4-inch silicon wafer was cleaned with permonosulphuric acid and demineralized water. Afterward, the wafer was spin-coated and dehydrated through a 15 min dehydration bake at 200 °C. An 800 nm thick layer of negative photoresist SU-8 (18% solid) was spin-coated and pre-baked at 65 °C for 1 min. Photolithography was performed with a laser beam 4-inch lithography mask (Deltamask, Netherlands). The exposure time was optimized for the SU-8 thickness, structure resolution, and lamp intensity of the mask aligner (MJB3, Süss MicroTec, Germany). Exposure time was set to 1.3 seconds in vacuum contact mode. Furthermore, a 5 min post bake at 65 °C and 95 °C was performed, and the wafer was developed in a negative resist developer (mrdev 600, micro resist technology GmbH, Germany). Afterward, a second SU-8 layer (52% solid) with a thickness of 10 µm was spin-coated onto the wafer. The lithography process was equal to the first layer except for the pre-bake process, which was set to 5 min at 65 °C and 5 min at 95 °C, and the exposure time was adjusted to 6 seconds.

The wafer was covered with PDMS in a ratio of 10:1 between the base and curing agent (Sylgard 184 Silicone Elastomer, Dow Corning Corporation, USA). Afterward, the wafer was degassed in an exicator for 30 min and backed at 80 °C for 2 hours (universal cupboard, Memmert GmbH, Germany). After this step, the PDMS chips were cut out from the wafer, cleaned three times with isopropanol, and blown dry with pressurized air. The cover glasses (D 263 T eco, 39.5x34.5x0.175 mm, Schott, Germany) for the microfluidic chip were also cleaned in this step. Afterward, the PDMS chip and the cover glass were oxygenized with O_2_ plasma (Femto Plasma Cleaner, Diener Electronics, Ebhausen, Germany) for 24 seconds with a power of 45% and assembled. Prior to the use, PDMS-glass bonding was strengthened by a 2 min bake at 80 °C.

# **Cell volume calculation**

Cell volume calculations were performed according to Dusny *et al.* [36]. Cell widths and lengths were extracted from time-lapse images based on the rod-shaped cell morphology of *E. coli* (see Figure S1). Cells were mathematically segmented into three sub-bodies, comprising a core cylinder and two half-spherical pole caps, and the respective volumes of the bodies were calculated and summed to obtain the approximated volumes of the individual cells.


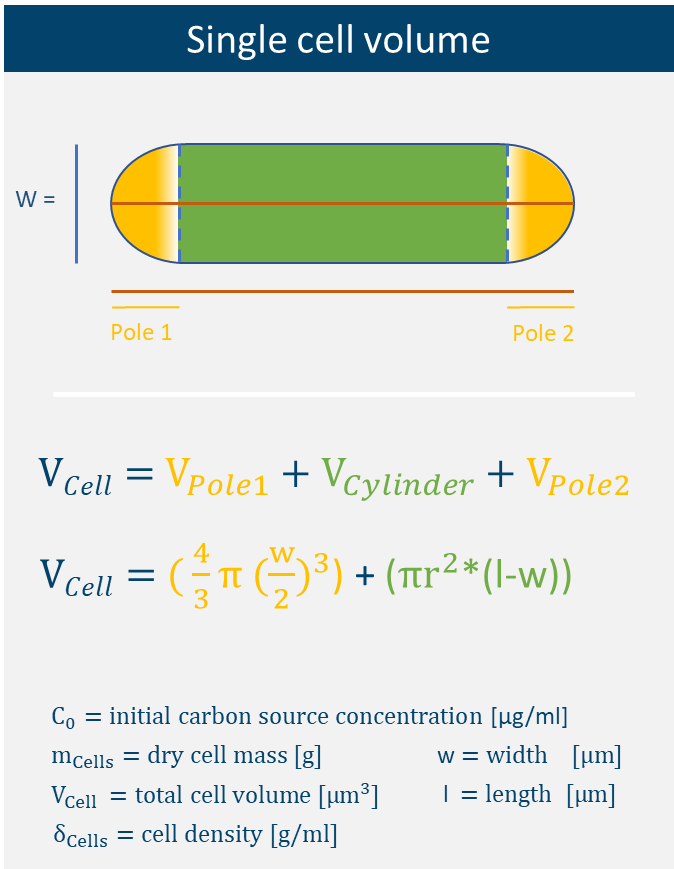


**Figure S1:** Morphometric cell volume calculation.

# **Transformation of *E. coli* EcLYS1 (pVWEx1-gfpUV)**

A single colony of *E. coli* MG1655 from a fresh LB-Kan50 agar plate was inoculated in sterile 200 ml LB-Tet10 overnight culture at 37 °C with vigorous aeration (200 rpm) (Ecotron, Infors HT, Germany) and harvested at OD_600_ between 0.4 0.5. Overnight cultures were cooled on ice and centrifuged in a pre-chilled rotor at 3900 rpm for 10 min at 4 °C. Cell pellets were resuspended in 500 mL ice-cold pure H_2_O and centrifuged at 3900 rpm for 5 min at 4 °C. Cell pellets were centrifuged under the same condition and resuspended in 10 ml volume with 15% glycerol. Final centrifugation and resuspension in 10 ml volume were carried out with 10% glycerol. 50µl aliquots of electrocompetent MG1655 cells were frozen with liquid N2 and stored at -80 °C until further use. Plasmid isolation of 2 ml *E. coli* EcLYS1 (pVWEx1-gfpUV) overnight culture was performed with NucleoSpin, Plasmid, Mini kit (Macherey-Nagel, Germany) according to the protocol. 50 µl of electrocompetent MG1655 cells were thawed on ice for 20 min and mixed with 50 - 70 ng of isolated pVWEx1-gfpUV plasmid DNA prior to electroporation. Electroporation was performed for 5 ms (2400U) (Eporator, Eppendorf, Germany), immediately suspended into 400 μl pre-warmed SOC medium and incubated (45 min at 37 °C, 300 rpm). The transformation suspension was centrifuged for 2 min at 7000 rpm. The supernatant was discarded, and the cell pellet was dissolved in 50 µl SOC medium, plated on LB agar in the presence of 50 μg mL^-1^ kanamycin, and incubated at 37 °C overnight. To ensure a successful transformation, a single colony of MG1655 (pVWEx1-gfpUV) of the LB overnight transformation plate was taken and transferred into 100 mL baffled shake flasks filled with 20 mL of sterile LB-Kan50 medium, grown to mid-exponential growth and induced with IPTG [1mM] final for GFP signal screening.
